# Mesenchymal chondrosarcoma of maxilla misdiagnosed as a benign fibrous lesion: A rare case report from Syria

**DOI:** 10.1016/j.ijscr.2025.110874

**Published:** 2025-01-12

**Authors:** Abdulrahman Abdo Shouman, Kamilla A. Mohammed, Hadeel M. Kaadeh, Yehia M. Haphian, Mohamad M. Hamzeh

**Affiliations:** a Faculty of Medicine, University of Aleppo, Aleppo, Syrian Arab Republic; b Faculty of Medicine, University of Damascus, Damascus, Syrian Arab Republic

**Keywords:** Neoplasm, Maxilla, Chondrosarcoma, Mesenchymal, Swelling

## Abstract

**Introduction:**

Mesenchymal chondrosarcoma (MC) is a high-grade variant of chondrosarcoma, essentially composed of poorly differentiated spindle cells interspersed with areas of cartilage or chondroid matrix. MC is extremely rare; it only accounts for 0.1 % of head and neck tumors and for only 1 % of all chondrosarcomas (CSs).

**Case presentation:**

A 21-year-old man presented with a medical history of a painful irritation at the dextral maxillary region, presented as a mass at the vestibule of the oral cavity near the upper molars, and had been misdiagnosed as a benign fibrous lesion and excised without performing a biopsy. Magnetic resonance imaging (MRI) revealed an invasive lesion filling the right maxillary sinus and penetrating the orbital floor. A biopsy was then performed and revealed an MC.

**Clinical discussion:**

The patient underwent a wide surgical resection, except for the infraorbital region, in which the tumor was surrounded by a fibrous capsule separating it from the anatomical structures of the eye. Due to the lack of wide resection in the orbital floor area (to preserve the eyeball), we applied the chemotherapy that was done with cisplatin and doxorubicin.

**Conclusion:**

Confirmed diagnosis by biopsy and treatment, both surgical and chemical, with frequent follow-up are decisive factors in progressing MC.

## Highlights

• Mesenchymal chondrosarcoma (MC) composed of chondroid matrix.
• MRI showed an invasive lesion that invades in the maxillary sinus
• The patient receive postoperative chemotherapy and place under clinical surveillance
• The outcomes ranging from tumor progression and metastasis to a tumor response

## 1 Introduction

Chondrosarcoma (CS) is an umbrella term for neoplasms that originate from cartilage. Several variants of CS have been proposed including: I-Conventional chondrosarcoma, which accounts for nearly 90 % of all chondrosarcomas, II-dedifferentiated chondrosarcoma, III-Clear-cell chondrosarcoma, IV-Mesenchymal chondrosarcoma, V-Juxtacortical chondrosarcoma, VI-Secondary chondrosarcoma.

According to cellularity, atypia, and pleomorphism, CS is classified into three histologic grades: grade I (low grade), grade II (intermediate grade), and grade III (high grade) [1].

Mesenchymal chondrosarcoma (MC) is a high-grade variant of chondrosarcoma, essentially composed of poorly differentiated spindle cells interspersed with areas of cartilage or chondroid matrix [2]. MC is extremely rare; it only accounts for 0.1 % of head and neck tumors and for only 1 % of all CS [3]. It occurs in an age group of between 30 and 60 years [4]. However, it should not be neglected in other age groups. No sex predilection is observed [1]. It is difficult to perform a truly representative biopsy of a chondrosarcoma due to the existence of different histologic grades in areas of the lesion. Identification of the most aggressive component of the tumor is critical [1]. The primary treatment for MC is wide surgical excision with negative margins. The prognosis of maxillary MC is poor due to the high late recurrence [5]. Metastasis may occur relatively late [6].

Here, an additional rare case of MC of the maxillary sinus in a 21-year-old male is reported and discussed. To the best of our knowledge, this is the first documented case mesenchymal chondrosarcoma of maxilla in Syria.

## 2 Methods

The work has been reported in line with the SCARE criteria [11].

### 2.1 Case presentation

**Fig. 1:**
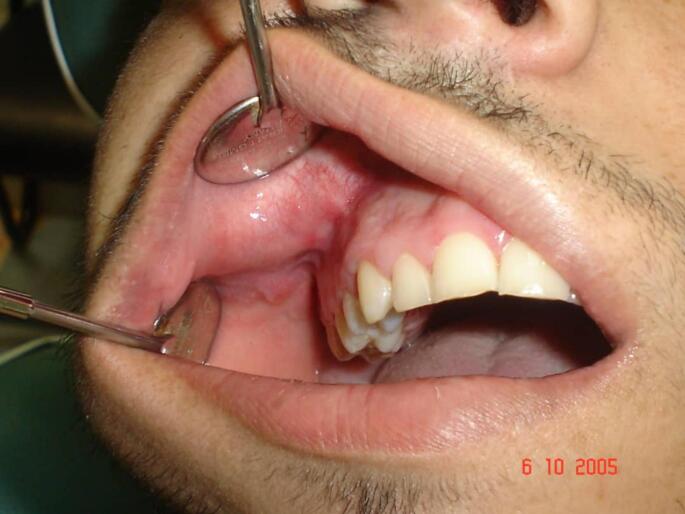
Intraoral photograph of the patient showing the lesion involving the vestibule of the oral cavity near the upper molars.

A 21-year-old man presented with a medical history of a painful irritation at the right maxillary region, which had occurred 8 months prior to our consulting, presented as a mass at the vestibule of the oral cavity near the upper molars (Fig. 1).

The lesion was misdiagnosed as a benign fibrous lesion and excised without performing a biopsy, it recurred with more pain, tooth movement, and diffuse swelling.

**Fig. 2:**
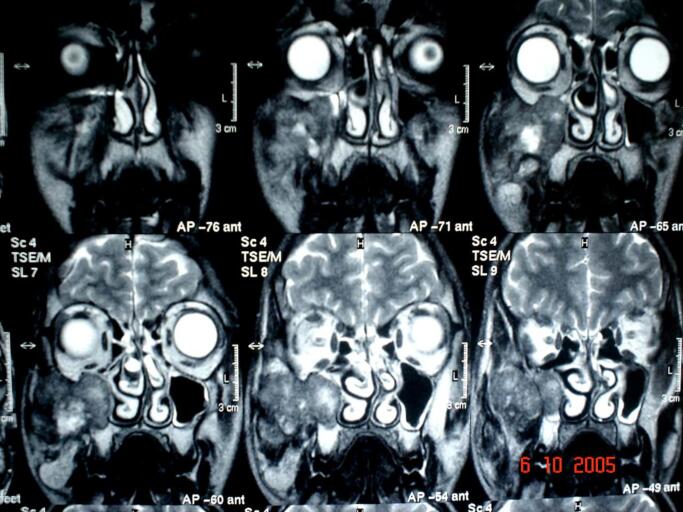
MRI (coronal view) showing invasive lesion which invades the sinus maxillaris and penetrates to the orbital floor and zygomatic bone.

Magnetic resonance imaging (MRI) revealed an invasive lesion in the right maxillary sinus which spread to the orbital floor and zygomatic bone (Fig. 2).

**Fig. 3:**
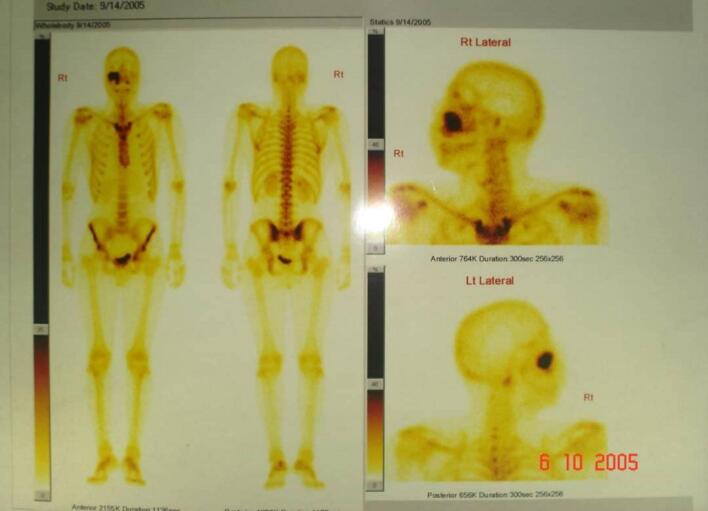
Bone scan image of the patient showing the tumor area and the absence of metastases.

A biopsy had been performed and revealed high cellularity, prominent nuclear atypia, and a marked increase in the number of mitotic figures; these features represent a high grade of mesenchymal chondrosarcoma. A bone scan was performed to investigate progress and tumor metastases and showed no metastases (Fig. 3).

**Fig. 4:**
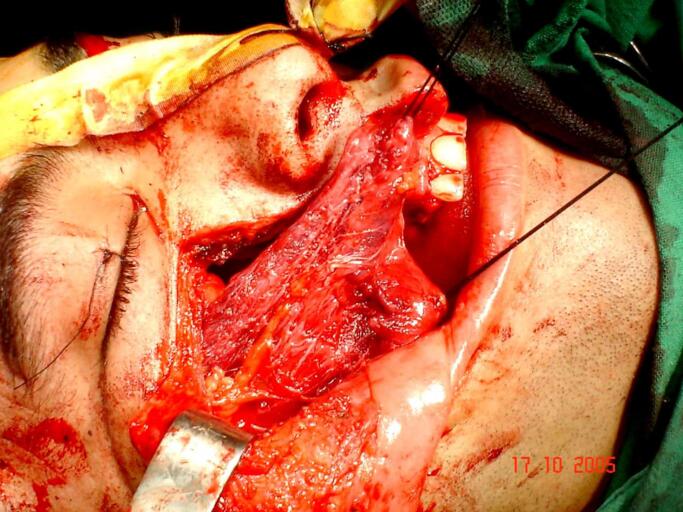
Hemimaxillectomy and closing the resection site with a muscle graft.

A right hemimaxillectomy had been performed, excising the zygomatic bone and the rest of the orbital floor (Fig. 4).

**Fig. 5:**
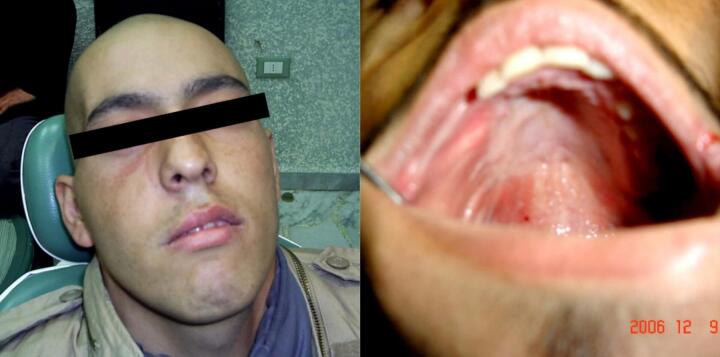
Postoperative extra and intra-oral photograph showing the surgical site.

Since the tumor was surrounded by a fibrous capsule separating it from the anatomical structures of the eye, the eye and surrounding structures were retained. The ablation was compensated and closed by a muscle graft from temporalis muscle (Fig. 5). The patient received postoperative chemotherapy and was then placed under clinical, radiological, and laboratory surveillance for five years in consecutive periods and periodically after another five years. Surveys were conducted once a year, and the surveillance lasted for ten years.

### 2.2 Clinical discussion

MC is an uncommon type of CS that was first described by Lichtenstein and Bernestein (1959) [2]. These tumors arise both from skeletal and extraskeletal sites, with a tendency toward the head and neck regions. The most common location for MC is the jaw bones. Orbit, meninges, nasal and sinus mucosa, and parapharyngeal space are the most common extraskeletal sites reported in the literature [7]. The clinical manifestations of MC include swelling, pain, nasal blockage, epistaxis, loose teeth, diplopia, and difficulty hearing, in order of their frequency. The duration of the symptoms ranges from one to 10 months [7].

. Evans et al. have classified CS into Grades I, II, and III based on mitotic rate, cellularity, and nuclear size [9]. Grade I lesions resemble benign cartilage, and they do not metastasize. Grade II lesions recur locally more often than grade I lesions and have a 10 % incidence of metastasis, which raises the incidence of grade III lesions up to 70 % [5]. In our case, although the tumor was high-grade, the bone scan was negative (the presence of metastases in other regions was not confirmed due to the unavailability of the PET scan), and therefore chemotherapy was administered to reduce the recurrence rate and eliminate metastases if they were present.

Histologically, the lesion must be differentiated from similar other lesions like hemangiopericytoma, Ewing's sarcoma, rhabdomyosarcoma, small-cell osteosarcoma, and malignant melanoma [4]. In our case, the misdiagnosis was made clinically, and a mass from the vestibule of the mouth was removed as benign fibrous tissue and was not sent for histopathology. The patient returned several months later with a story of a painful swelling in the right cheek with tooth mobility. There are no classical radiographic features of MC. Radiographically, these lesions appear as osteolytic, radiolucent shadows with ill-defined or ragged borders [10]. The most frequent radiographic feature is aggressive medullary bone destruction, with a moth-eaten to permeative pattern [3]. In our case, an MRI was performed that showed an invasive lesion filling the right maxillary sinus and penetrating the orbital floor. The primary therapeutic modality for MC is wide surgical excision with a tumor-free margin of 2 to 3 cm [3]. In our case, a wide surgical resection was performed, except for the infraorbital region, in which the tumor was surrounded by a fibrous capsule separating it from the anatomical structures of the eye. Due to the lack of wide resection in the orbital floor area (to preserve the eyeball), we applied the chemotherapy that was done with cisplatin and doxorubicin. The prognosis of MC after treatment is generally poor because the lesion has a predilection for late recurrence, either locally or metastatically [3]. The outcomes that can occur after treatment are variable, ranging from rapid tumor progression and metastasis (the worst-case scenario) to a complete tumor response (the best-case scenario), which resembles our case because of chemotherapy in our estimation [3]. Common sites of metastasis are the lung, bone, and lymph nodes. However, local recurrence usually occurs before distant metastases [7]. In our case, the patient underwent clinical, laboratory, and radiological monitoring for 5 years as a first phase during successive periods, then once a year in the following 5 years, and the next evaluation was performed after 9 years, and he was well. Local recurrence and distant metastases can occur many years after treatment, making long-term follow-up essential [7].

## 3 Conclusion

It is important to consider MC in a patient who presents with the same previous symptoms. A biopsy must also be taken for suspicious lesions to avoid misdiagnosis, as in our case. Chemotherapy should be considered as a treatment option after surgical resection. Follow-up is important to find out recurrence and improve the prognosis.

## CRediT authorship contribution statement

All authors were both involved in the conception and coordination of this report and drafted the manuscript. Additionally, all authors have read and approved the final version.

## Consent

Informed verbal consent was obtained from the patient for publication of this case report and any accompanying images. This consent is sufficient in our country.

## Ethical approval

This study is exempt from ethical approval in our institution (University of Aleppo, Aleppo, Syria and University of Damascus, Damascus, Syria) because the content of the case report does not require ethical approval.

## Guarantor

Abdulrahman Abdo Shouman. Mohamad Mahmoud Hamzeh.

## Sources of funding

This research did not receive any specific grant from funding agencies in the public, commercial, or not-for-profit sectors.

## Registration of research studies

Not applicable.

## Declaration of competing interest

No conflicts of interest.
